# Antioxidant potential and essential oil properties of *Hypericumperforatum* L. assessed by application of selenite and nano-selenium

**DOI:** 10.1038/s41598-022-10109-y

**Published:** 2022-04-13

**Authors:** Mahmonir Rezaei Nazari, Vahid Abdossi, Fariba Zamani Hargalani, Kambiz Larijani

**Affiliations:** 1 Department of Horticultural Sciences, Science and Research Branch, Islamic Azad University, Tehran, Iran; 2 Department of Natural Resources and Environmental, Science and Research Branch, Islamic Azad University, Tehran, Iran; 3 Department of Chemistry, Science and Research Branch, Islamic Azad University, Tehran, Iran

**Keywords:** Plant physiology, Secondary metabolism, Enzymes

## Abstract

It is necessary to develop a simple way to achieve food quality quantitatively. Nanotechnology is a key advanced technology enabling contribution, development, and sustainable impact on food, medicine, and agriculture. In terms of medicinal and therapeutic properties, *Hypericum* *perforatum* is an important species. For this study, a randomized complete block design with three replications was used in each experimental unit. The foliar application of selenite and nano-selenium (6, 8, 10, and 12 mg/l), control (distilled water), at the rosette stage and harvesting at 50% flowering stage has been applied as an alleviation strategy subjected to producing essential oils and antioxidant activity. Experimental results revealed that the selenite and nano selenium fertilizers had a significant effect on traits such as total weight of biomass, essential oil percentage, the content of hypericin and hyperforin, the selenium accumulation in the plant, relative leaf water content, chlorophylls, phenolic content, proline, catalase, peroxidase, malondialdehyde, and DPPH. The highest essential oil content was obtained from the control treatment when the accumulation of selenium was achieved with 12 mg/l nano-selenium. The maximum rate of hypericin was seen in the foliar application of 8 mg/l selenite whereas the maximum hyperforin was gained at 10 mg/l selenium. Conceding that the goal is to produce high hypericin/ hyperforin, and also the accumulation of selenium in the plant, treatments of 6 and 8 mg/l of selenite and nano-selenium could be applied. Consequently, an easy detection technique proposed herein can be successfully used in different ranges, including biology, medicine, and the food industry.

## Introduction

*Hypericum* *perforatum* belongs to the Hypericaceae family that has more than 469 species in the world^1^, of which 17^2^ to 19 species^3^ have been reported in Iran. *St.* *John's* *wort* is a perennial plant and the most important medicinal species of this genus. It is a branched plant 40–80 cm high^4,5^. In the upper parts of *H.* *perforatum*, several yellow flowers are produced, which are usually 1 to 2 cm wide. Flowers produced in late summer have capsules that contain tiny brown seeds. Although this plant is native to Europe, it is distributed in all temperate regions in Asia, Australia, and North and South America^6^. In terms of medicinal and therapeutic properties, *H.* *perforatum* is the most important species of this genus, and today it is widely used in the treatment of mild-to-moderate depression. The history of the traditional use of this medicinal plant dates back to over 2000 years ago. Antidepressant, anti-inflammatory, antimicrobial, antiviral, and anti-cancer properties have been reported for *H.* *perforatum.* Various studies have reported that *H.* *perforatum* has antiviral^7^, wound healing^8^, antioxidant^9^, antimicrobial^10^, and antibacterial properties^11^. Clinical trials have been conducted since the early 1990s on the effect of hypericin on the treatment of viral diseases, and experiments in this area show that the plant with low toxicity has significant effects against HIV-like viruses. So far, a wide range of biologically active compounds has been identified and reported in this plant, including hypericin and hyperforin. Chemical research on the components of *H.* *perforatum* has led to the identification of several groups of pharmaceutically active compounds. The most important groups include naphthodianthrones (hypericin and pseudo-hypericin), phloroglucinols (hyperforin and adhyperforin), xanthones and flavonoids (such as phenylpropanes, flavanol glycosides and bioflavonoids) as well as essential oils. Various biological activities including wound healing, anti-anxiety and seizures, antiviral, antifungal, and antioxidant properties have been attributed to the compounds in the extract and essential oil of various species of the genus Hypericum. There are various reports on the antimicrobial activity of essential oils of different species of the genus Hypericum against bacteria and fungi of human and plant pathogens^12^. Yaman et al.^13^ reported the stem exhibited the stronger antioxidant activities than other samples, and vanillic acid, ferulic acid and gallic acid could be produced by in vitro culture of Hypericum.

Selenium is one of the essential elements in the human diet. Although selenium has a variety of functions, its antioxidant and anti-cancer properties are of particular importance to humans. Selenium deficiency in the human diet also causes stunted growth, and thyroid dysfunction; particular cardiomyopathy, myocardial ischemia/infarction^14^, infectious diseases caused by viruses e.g., HIV, IAV, hepatitis C virus, poliovirus, West Nile virus^15^. Selenium concentrations are usually higher in younger leaves than in older leaves during seedling growth^16^. Contrary to selenate (Se^+6^) which enters plant cells through sulfate carriers, selenite (Se^+4^) is accumulated by plants via phosphate pathway^17^. Selenium-enriched production is one of the most effective ways to deal with the deficiency of selenium. For example, Finland started to add Se in fertilizer in 1984 to reduce the occurrence of Se deficiency in the population, through a policy established by the Ministry of Agriculture and Forestry^18^.

The interference study shows that it did not interfere with the determination of Se at their 15 respective tolerable limits. The enrichment contents of Se at an ultra-trace level in natural water, agricultural soil and food samples were determined successfully by GF-AAS. The application of factorial design for the screening of extraction parameters shows that concentration of APDC, pH and vortex mixing time is the most significant factor for maximum recovery of Se. The optimum values of all parameters were used for the enrichment of trace level Se in natural water, agricultural soil and food samples^19^. In another study Ali et al.^20^ stated the interaction between Arsenic (As) and Selenium is a critical factor for a detailed systematic understanding of the transportation, environmental fate, and associated toxicological effects of these metalloids in plants, animals, and humans. They emphasized the imbalance of Se compounds can lead to the generation of ROS, which can inhibit or decrease genomic stability. The Arsenic and Se nexus also affect cellular signaling through activation of transcription. The developed method is more selective, sensitive, reliable, and efficient for accurately assessing low level of selenium food and soft drink samples^21^.

Nanoparticles react with plants to cause a variety of physical and physiological changes, which significantly depend on the properties of the nanoparticles. The effectiveness of nanoparticles depends on their concentration and varies from plant to plant. The efficiency of nanoparticles depends on the chemical composition, size, surface area, reactivity, and concentration at which they respond positively. Nanoparticles have positive and negative effects on plant growth^22^. Irmak^23^ conducted a study to investigate the effect of selenium application on plant growth and some quality parameters in Peanut (*Arachis* *hypogaea*).

Se deficient has been considered in some developed countries and achieved some success by introducing selenium into their native plants, but this problem has been less addressed and almost no action has been taken in this regard. Since different physiological traits related to selenium (Se) have not been discussed in detail on *H.* *perforatum*, the aim of this study was to evaluate the selenium-enrichment along with phytomorphological, essential oil, (Se) accumulation and enzymatic activity. Furthermore, we pointed to identify the appropriate concentration of Selenium that can develop essential oil and other features.

## Methods

**Figure 1:**
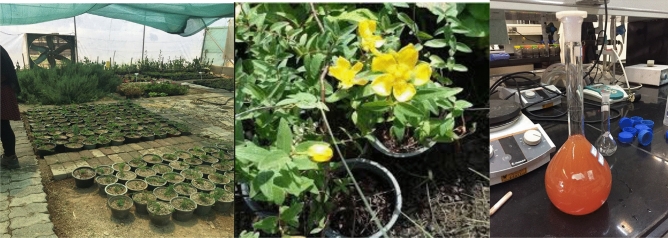
The stages of this research.

The study aimed to survey the effect of selenium and nano-selenium on vegetative growth and phytochemical and enzymatic properties of *St.* *John's* *wort* in the Tehran Municipality greenhouse in 2020. This experiment was conducted with three replications in a randomized complete block design. For this purpose, nano-selenium at concentrations of 6, 8, 10, and 12 mg/l were foliar applied simultaneously with the foliar application of sodium selenite at the same concentration. Foliar spraying of distilled water was used as a control (Fig. 1).

One-year-old seedlings of Topaz cultivar were obtained from Pakan Bazr Isfahan Company and planted in pots in March. After seedling establishment, foliar application was performed. The specimen was presented in the central herbarium of Tehran University (Herbarium Code: TUH). (6398 No). Samples were authenticated by a botanist Dr. Moradi^24^. Data collection and plant harvesting were performed at 50% flowering stage.

Plant height, root length inflorescence length was measured using the scaled ruler. The number of inflorescences is noted per each plant. The shoot weight, root weight, total biomass weight, inflorescence each pot was milled from the soil surface and their fresh weight was immediately measured by a digital scale with 0.01 g accuracy^25^.

After grinding the flowers and leaves, their essential oils were separately collected by water distillation using a Clevenger (Schott-DURAN, Germany) from the plants treated in FC = 25% (severe tension). Gas chromatography-mass spectrometry (GC/MS: Shimadz, Japon) was applied to separate and identify essential oil substances. GC/MS analysis was conducted on GC detector coupled with a Hewlett Packard 6890 gas chromatograph, equipped with a cross-linked 5% Phenyl di methyl siloxane HP-5MS capillary column (30 m × 0.25 mm, film thickness 0.32 μm). GC operating conditions were as follows: Carrier gas, helium with a flow rate of 1 ml/min; column temperature, 60˚-220ºC at 3ºC/min; injector and detector temperatures, 250ºC; volume injected, 0.1 μl of the oil; split ratio, 1:20. HP- 5973 MS operating parameters were as follows: Ionization potential, 70 eV; ion source temperature, 230ºC. Each object and interpretation constituent of essential oils were identified by GC retention indices relative hypericin’ and KI computer matching with the library^26^.

For measurement of selenium content, initially 1 g from dried petals samples were digested in 5 mL mixture of nitric acid and concentrated perchloric acid (with volume rate of 4:1) at temperature 130 °C for an hour. After cooling, 5 ml of concentrated hydrochloric acid were added and heated for 20 min at a temperature of 115 °C. Finally, atomic spectrometry (ICP-OES spectrometer Integra XL2, GBC Australia) was used to determine of selenium contents^27^.

The relative water content (RWC) of the leaves was estimated as described by^28^ and calculated according to RWC = [(FW D DW)/(TW D DW)] × 100, where FW is the fresh weight of the leaves, TW is he weight at full turgor, measured after floating the leaves for 24 h on water in the light at room temperature, and DW is the weight estimated after drying the samples for at least 4 h at 80 ºC or until a constant weight is achieved.

Chlorophyll content was measured as explained. 0.5 g leaf was calculated. The material was homogenized in a homogenizer by the addition of 10 ml of 80% acetone. The acetone extract containing all chloroplast was centrifuged at 2500 rpm for 5 min. This obtained extract was diluted by adding 9 ml of 80% acetone / ml of the extract. It was read on a spectrophotometer at 645 nm and 663 nm^29^.

Phenolic content 0.1 ml of sample/standard solution in 5% methanol was mixed with 1 mL Folin–Ciocalteu reagent diluted with water and 1 mL 10% Na_2_CO_3_ was added. The absolute absorbance was taken at 760 nm^30^.

To obtain the methanolic extract, 100 g of dry samples were soaked in the methanol for 48 h. The capacity of extracts from to decrease the radical 2,2-diphenyl-1-picrylhydrazyl (DPPH) was evaluated using the method of Atmani et al.^31^. The 15 ml of a solution of DPPH in methanol (5 mM) were mixed with 2.45 ml of a solution of plant extract for 30 min and the absorbance was recorded at 517 nm. The normal purple color of DPPH will turn into yellow when its singlet electron is paired with a hydrogen atom coming from a potential antioxidant. The scavenging activity of extracts was measured as percent scavenging = [*A*0-(*A*1–*A*S)]/*A*0]100, where *A*0 is the absorbance of DPPH alone, *A*1 is the absorbance of DPPH + extract and *A*S is the absorbance of the extract only. The results were expressed as IC50 (The amount of exact to scavenge 50% of DPPH). Fresh tissue (100 mg) was homogenized, and 3 ml of 50 mM Na-phosphate buffer (pH 7.0) (including 2 mM-dithiothreitol, 2 mM EDTA, 0.2 percent triton x-100, 50 mM tris–HCl, and 2 percent polyvinylpyrrolidone) was added to make the enzymatic extract. The resultant extract was centrifuged for 30 min at 14,000 rpm.

The CAT activity (EC 1.11.1.6) was determined using the Aebi^32^ technique of determining H_2_O_2_ uptake. A spectrophotometer (Model U-1800, Tokyo, Japan) was used to quantify the quantity of H_2_O_2_ in the reaction mixture after one minute at 240 nm using the extinction coefficient (= 0.28 mMol^−1^ cm^−1^).

Guaiacol was used to test peroxidase (POX) activity (EC 1.11.1.7). A spectrophotometer was used to measure the adsorption rate of tetraguaiacol (due to guaiacol oxidation) at 470 nm at the start of the reaction and after one minute. The quantity of tetraguaiacol generated was determined using the absorbance changes and the extinction coefficient of tetraguaiacol (= 25.5 mMol^−1^ cm^−1^)^33^.

Free proline content as assessed in leaf tissue by the ninhydrin method of Bates et al.^34^. Fresh leaf material (0.5 g) was stirred with 5 ml distilled water and boiled in a water bath for 30 min. After centrifugation samples were filtered through paper and 1 ml of the supernatant was mixed with 2 ml freshly prepared ninhydrin reagent (0.5 g ninhydrin in 50 ml of 60% acetic acid). The color reaction developed after incubation of the samples for 1 h in a boiling water bath. Registration was performed spectrophotometrically after toluene extraction, and estimation of proline concentration was based on a previously prepared standard curve. The equivalent dry weight was obtained by drying an identical leaf quantity at 80 ºC to a constant weight.

The Madhava and Sresty^35^ technique was applied to determine membrane lipid peroxidation, using MDA measurement as the ultimate result of membrane lipid peroxidation. The quantity of MDA was estimated using a spectrophotometer to measure the absorbance at 532 nm and 600 nm (Model U-1800, Tokyo, Japan). All methods were performed by following the relevant guidelines/regulations/legislation.

## Results

The results of the analysis of variance showed that the effect of selenite and nano selenium fertilizers on plant height, inflorescence length/number/weight, shoot weight, root length, total biomass weight, essential oil percentage, and hypericin and hyperforin content was significant at 1% level, and on the root weight at 5% level (Table 1).

**Table 1:** Effect of selenium and nano-selenium on morphophysiological characteristics and growth of *St.* *John's* *wort* using analysis of variance.

S.O.V	df	Plant height (cm)	Inflorescence length (cm)	Number of inflorescences	Inflorescence weight (g/plant)	Shoot weight (g/plant)	Root length (cm)	Root weigh (g/plant)	Total biomass weight (g/plant)	Essential oil percentage (%)	Hypericin content (mg/g dry matter)	Hyperforin content (mg/g dry matter)
Replication	2	75.69*	26.01 ns	9 ns	57.76**	147.21**	87.84**	2.25 ns	36.4*	0.00 ns	0.75**	73.38**
Treatment	8	127.05**	35.45**	54**	45.20**	238.58**	39.45**	37.77*	773.08**	0.00**	3.53**	267.75**
Error	16	7.22	8.52	5.25	5.58	2.69	0.62	5.46	10.59	0.00	0.06	2.19
%CV		5.52	13.59	22.91	5.35	2.90	3.4	13.03	3.66	23.06	10.82	3.60

S.O.V	df	Selenium (µg/g DW)	RWC (%)	Chlorophylls *a* (g/100 g FM)	Chlorophylls *b* (g/100FM)	Total chlorophylls (g/100 g FW)	Phenolics (mg/l FW extract)	Proline (µmol/g FW)	CAT (U/g FW/min)	POD (U/g FW/min)	MDA (mM/d FW)	DPPH (%)
Replication	2	378.56**	272.03**	0.19**	0.006**	0.26**	2.08**	44.44**	0.002**	0.40*	3321.6*	54.76**
Treatment	8	16,804.1**	508.77**	0.09**	0.02**	0.17**	3.07**	614.33**	0.004**	0.61**	201,147**	350.08**
Error	16	21.81	0.39	0.00	0.00	0.00	0.29	0.98	0.00	0.07	769.53	4.59
%CV		3.84	3.88	4.00	7.04	3.50	13.06	3.62	13.48	15.14	9.28	3.83

ns, * and ** indicate non-significance and significance at the level of 5 and 1%, respectively.

*RWC* relative water content, *CTA* catalase, *POD* peroxidase, *MDA* malondialdehyde.

ns, * and ** indicate non-significance and significance at the level of 5 and 1%, respectively.

Comparison of means showed that the highest plant height was obtained from control treatment (58 cm) and foliar application of 6 mg/l selenite (56.4 cm) (Table 2). Inflorescence length was the highest in the control treatment and two treatment levels of 6 and 8 mg/l selenite and nano-selenium (Table 2). The number of inflorescences was the highest in the control treatment and the first treatment level (6 mg/l) of selenite and nano-selenium (Table 2).

**Table 2:** Comparison of means of the effect of selenium and nano-selenium on morphophysiological characteristics and growth of *St.* *John's* *wort.*

	Plant height	Inflorescence length	Number of inflorescences	Inflorescence weight	Shoot weight	Root length	Root weight	Total biomass weight	Essential oil percentage	Hypericin content	Hyperforin content
	cm	cm		g/plant	g/plant	cm	g/plant	g/plant	%	mg/g dry matter	mg/g dry matter
Co	58a	25.5a	16a	21a	70a	27a	24a	115a	0.14a	2.7cd	44c
si6	56.4a	25ab	14ab	17b	66b	26ab	22ab	105b	0.1b	3.2b	50b
8	50bc	24ab	10cd	15c	60c	25bc	19bc	94cd	0.09bc	3.8a	44c
10	48.5bc	20bcd	8de	12d	55d	22d	17.5c	84.5e	0.07bcde	1.5e	57a
12	41de	18cd	6ef	10e	47e	20ef	16.5c	73.5f	0.05de	1f	33e
Nanosi6	51.5b	23.8ab	14ab	17.5b	62c	25bc	18bc	97.5c	0.1b	3bc	46c
8	49b	22.5abc	12bc	15c	57d	23.8c	17.5c	89.5de	0.08bcd	2.5d	41d
10	45.6cd	18.5cd	6ef	12d	48e	21de	15cd	75f	0.06cde	1.2ef	34e
12	38e	16d	4f	9e	44f	18.8f	12d	65g	0.04e	0.8f	26f

	Selenium (µg/g DW)	*RWC* (%)	Chlorophylls *a* (g/100 g FM)	Chlorophylls *b* (g/100 g FM)	Total chlorophylls (g/100 g FM)	Phenolics (mg/l FW extract)	Proline (µmol/g FW)	CAT ( U/g FW/min)	POD (U/g FW/min)	MDA (mM/day FW)	DPPH (%)
co	37h	84a	0.77e	0.26d	1.03e	3.5c	12h	0.05e	1.1d	68d	35f
si6	49.5g	76b	0.96d	0.3cd	1.26d	3.23c	15g	0.07de	1.3d	92d	47e
8	79f	75b	1.02d	0.32bc	1.34d	3.31c	25d	0.07de	2ab	103d	56c
10	120d	70c	1.13c	0.35b	1.48c	3.28c	33c	0.1bc	2.3a	185c	68a
12	137c	61f	1.23b	0.28cd	1.51c	4.54b	48b	0.15a	1.5cd	590b	61b
Nanosi6	94e	75.5b	1d	0.32bc	1.32d	3.41c	17f	0.08cd	1.4cd	110d	52d
8	122.63d	68.4d	1.19bc	0.36b	1.55c	4.66b	19e	0.09cd	1.8bc	222c	71a
10	167.52b	66e	1.25ab	0.46a	1.71b	5.2ab	26d	0.12b	2.4a	695a	57c
12	287.52a	58g	1.32a	0.49a	1.81a	6a	52a	0.16a	1.5cd	624b	55cd

The same letters in each column indicate that there is no significant difference between the means.

*RWC* relative water content, *CTA* catalase, *POD* peroxidase, *MDA* malondialdehyde.

The same letters in each column indicate that there is no significant difference between the means.

The highest inflorescence weight (21 g/plant), shoot weight (70 g/plant), root weight (27 g/plant), total biomass weight (115 g/plant), and essential oil content (0.14%) was obtained from the control treatment. The highest root weight was obtained from the control treatment (24 cm) and foliar application of 6 mg/l selenite (22 cm). The highest accumulation of hypericin (3.8 mg/g dry matter) was obtained from the foliar application of 8 mg/l selenium. The highest accumulation of hyperforin (57 mg/g dry matter) was obtained from the foliar application of 10 mg/l selenite.

The effect of selenite and nano-selenium on the amount of selenium accumulation in the plant, relative leaf water content, the content of chlorophylls *a*, *b*, and total, the phenolic content and proline content, and production and accumulation of catalase, peroxidase, malondialdehyde, and DPPH enzymes were statistically significant at the 1% level (Table 1).

It was observed that the highest accumulation of selenite (287.52 μg/g dry matter), the highest content of chlorophyll *a* (1.32 g), chlorophyll *b* (0.49 g) total chlorophyll (1.81 g), phenolic content (6 mg), and proline (52 μmol/g) were obtained in the foliar application of 12 mg/l nano-selenium. The highest catalase content was obtained from the foliar application of 12 mg/l nano-selenium (0.16 μ/g FW/min) and foliar application of 12 mg/l selenite (0.15 μ/g FW/min). Peroxidase was higher in the foliar application of 8 and 10 mg/l selenite and 10 mg/l nano-selenium than other treatments. The highest malondialdehyde leakage (695 μ/g FW/min) was obtained from the 10 mg/l nano-selenium treatment. The highest levels of antioxidant enzyme (71 and 68%) were obtained from the foliar application of 8 mg/l selenite and 10 mg/l nano-selenium, respectively.

The study of simple relationships between traits showed a significant positive correlation between the plant height and inflorescence length, number of inflorescences, inflorescence weight, shoot weight, root length, root weight, total biomass weight, essential oil percentage, the content of hypericin and hyperforin, relative water content, and peroxidase at the level of 1%. The plant height was also significantly correlated with the amount of selenite, chlorophyll *a,* *b*, and total chlorophyll, phenolic content, proline, catalase, malondialdehyde, and DPPH (Table 3). Similar to the plant height, the inflorescence length, number of inflorescences, inflorescence weight, shoot weight, root length, root weight, essential oil percentage, and amount of hypericin and hyperforin had a significant correlation with other traits (Table 3).

**Table 3:** Simple correlation of the effects of selenium and nano-selenium on morphophysiological characteristics and growth of *St.* *John's* *wort.*

	Plant height	Inflorescence length	Number of inflorescences	Inflorescence weight	Shoot weight	Root length	Root weight	Total biomass weight	Essential oil percentage	Hypericin content	Hyperforin content
	1	2	3	4	5	6	7	8	9	10	11
1	1.00										
2	0.83**	1.00									
3	0.84**	0.77**	1.00								
4	0.55**	0.45*	0.64**	1.00							
5	0.91**	0.85**	0.87**	0.57**	1.00						
6	0.90**	0.81**	0.79**	0.35 ns	0.93**	1.00					
7	0.71**	0.57**	0.71**	0.61**	0.82**	0.68**	1.00				
8	0.86**	0.77**	0.87**	0.76**	0.95**	0.81**	0.90**	1.00			
9	0.80**	0.81**	0.79**	0.62**	0.89**	0.78**	0.76**	0.89**	1		
10	0.77**	0.82	0.75**	0.52**	0.83**	0.82**	0.62**	0.79**	0.73**	1.00	
11	0.71**	0.59**	0.54**	0.31 ns	0.70**	0.63**	0.56**	0.64**	0.56**	0.58**	1.00
12	−0.78**	−0.68**	−0.75**	−0.74**	−0.79**	−0.66**	−0.81**	−0.87**	−0.74**	−0.73**	−0.68**
13	0.91**	0.78**	0.82**	0.60**	0.96**	0.90**	0.79**	0.93**	0.86**	0.79**	0.70**
14	−0.47*	−0.36 ns	−0.50*	−0.94**	−0.49*	−0.25 ns	−0.60**	−0.70**	−0.59**	−0.44*	−0.32 ns
15	−0.48*	−0.39*	−0.55**	−0.65**	−0.50*	−0.34 ns	−0.62**	−0.63**	−0.46*	−0.44*	−0.43*
16	−0.50*	−0.39*	−0.54**	−0.91**	−0.53**	−0.29 ns	−0.64**	−0.72**	−0.59**	−0.47*	−0.37*
17	−0.56**	−0.53**	−0.53**	−0.67**	−0.53**	−0.34 ns	−0.59**	−0.64**	-0.56**	-0.58**	-0.66**
18	−0.78**	−0.67**	−0.78**	−0.84**	−0.76**	−0.64**	−0.66**	−0.84**	−0.75**	−0.72**	−0.54*
19	−0.71**	−0.54**	−0.71**	−0.87**	−0.68**	−0.52*	−0.67**	−0.81**	−0.72**	−0.71**	−0.56*
20	−0.17 ns	−0.24 ns	−0.43*	−0.51*	−0.24 ns	−0.06 ns	−0.19 ns	−0.33 ns	−0.35 ns	−0.15 ns	0.16 ns
21	−0.73**	−0.72**	−0.76**	−0.72**	−0.82**	−0.67**	−0.69**	−0.86**	−0.73**	−0.81**	−0.76**
22	−0.39*	−0.33 ns	−0.42*	−0.63**	−0.45*	−0.28 ns	−0.48*	−0.56*	−0.56*	−0.28 ns	0.04 ns

	Selenium	*RWC*	Chlorophylls *a*	Chlorophylls *b*	Chlorophylls total	Phenolic content	Proline	*CAT*	*POD*	*MDA*	*DPPH*
	12	13	14	15	16	17	18	19	20	21	22
12	1.00										
13	−0.80**	1.00									
14	0.73**	−0.53**	1								
15	0.83**	−0.49*	0.71**	1							
16	0.8**	−0.55*	0.98**	0.84**	1						
17	0.8**	−0.52*	0.78**	0.73**	0.81**	1					
18	0.81**	−0.77**	0.69**	0.46*	0.66**	0.59**	1				
19	0.83**	−0.71**	0.84**	0.63**	0.83**	0.78**	0.87**	1			
20	0.26 ns	−0.15 ns	0.52*	0.51*	0.55**	0.25 ns	0.19 ns	0.28 ns	1		
21	0.8**	−0.78**	0.7**	0.64*	0.72**	0.77**	0.71**	0.82**	0.29 ns	1	
22	0.38*	−0.49*	0.68*	0.33 ns	0.61**	0.31 ns	0.41*	0.44*	060**	0.29 ns	1

ns, * and ** indicate non-significance and significance at the level of 5 and 1%, respectively.

*RWC* relative water content, *CTA* catalase, *POD* peroxidase, *MDA* malondialdehyde.

ns, * and ** indicate non-significance and significance at the level of 5 and 1%, respectively.

It was observed that the content of selenium had a significant negative correlation with morphological traits and yield of different organs and a significant positive correlation with the number of enzymes, chlorophylls, phenolic content, and proline. In contrast to selenium, relative water content had a significant positive correlation with agronomic traits and yields and a significant negative correlation with the number of enzymes, chlorophylls, phenolic content, and proline.

As can be seen in Table 3, physiological traits such as chlorophylls, enzymes, phenolic content, and proline had a significant positive correlation with each other.

## Discussion

Considering climate change^36^, greenhouse gases phenomenon^37^, and drought^38^ in the regions of the world and especially in Iran, introducing new crops with economic value and high water use efficiencies such as *H.* *perforatum* so the study of its response to different treatment levels is one of the important research priorities. The significant difference between replications was because of uneven greenhouse conditions, showing that the random complete blocks design was correctly selected. Moreover, the significant differences between traits (Table 1) show the selection of the hypothesis, the subject of research, and treatments, and the importance of research on *H.* *perforatum* and the introduced treatments.

Comparison of means showed that the highest plant height was obtained from control treatment and foliar application of 6 mg/l selenium (Table 2), and the plant height decreased with increasing the concentration of treatments. The decreased height with selenium treatments indicates the intolerance of *H.* *perforatum* to selenium stress. Because the number of inflorescences is affected by environmental and genetic factors and tillers, so by applying environmental treatments, the role of each of the environmental and genetic factors can be revealed. In this study, it was observed that inflorescences are more affected by environmental factors. The results of the effect of selenium as an environmental factor on the number of tillers are shown in Table 2 which are consistent with the findings Soleimani et al.^39^ and Uddin et al.^40^ on the effect of environmental and genetic factors on the number of tillers. The highest shoot weight, root weight, and total biomass weight were obtained from the control treatment. Therefore, the results of this part of the research show that *H.* *perforatum* is intolerant to selenite and nano-selenium.

The studied plant appears to cope with selenium and nano-selenium stress with changes in morphological traits^41^, decrease in photosynthetic and transpiration organs, increase in regulatory osmolytes^42^, changes in the synthesis of materials within the plant^43^, increased water-absorbing organs^44^ and finally allocation of photosynthetic material for active water absorption by energy^45^, all of which will reduce plant shoot yield as occurred for *H.* *perforatum*. The highest essential oil percentage was obtained from the control treatment, while the content of hypericin and hyperforin increased at low levels of selenium and nano-sellenium.

Basically, in plants, when they are stressed, the pentose phosphate pathway becomes more active and the production of essential oil increases. It has also been reported that plants under conditions of severe stress increase the percentage of other regulating compounds and osmolytes such as proline instead of essential oils and this is more evident in sensitive plants. The highest accumulation of hypericin and hyperforin was obtained from the foliar application of 8 mg/l and 10 mg/l selenite, respectively, showing that the use of small amounts of these two compounds can be useful for producing high-quality plants. Catalase enzyme was highest in the foliar application of 12 mg/l nano selenium and the foliar application of 12 mg/l selenite, which indicates the production of this enzyme at high levels of selenite and nano selenium. However, peroxidase production is faster than catalase in the plant, and it seems that at the same time as peroxidase production, malondialdehyde leakage reaches its maximum.

The results showed that in the foliar application of 12 mg/l nano-selenium, the maximum accumulation of selenium and the highest amount of chlorophyll a, chlorophyll b, total chlorophyll, phenolic content, and proline were obtained. Therefore, *St.* *John's* *wort* was a plant that absorbs selenium and nano-selenium, and despite the increase in chlorophyll content, a decrease in yield and an increase in antioxidant activity were observed. In general, the effect of selenite and nano-selenium particles depends on the composition, size, surface area, reactivity, and concentration in which they respond positively and have positive and negative effects on plant growth and development^22^. In our study, the same positive and negative results were observed on different traits, and in this regard, the results are consistent with the research of Ekinci et al.^22^. The results of this study are similar to the research on Lemongrass (*Melissa* *officinalis* L.) in terms of decreasing yield and increasing the concentration of chlorophyll a and b and the activity of catalase and malondialdehyde in the 10 mg/l treatment. Simple relationships between traits showed a significant positive correlation between morphological traits and yields. On the other hand, Selenium is of special interest in different research fields due to its narrow range between beneficial and toxic effects. Nanoparticles based on Se (SeNPs) are less toxic than inorganic and organic Se. The latest results on the elects of Se supplementation on metal-exposed plants indicate that coprecipitation of Se and metals in soil plays a key role in inhibiting the uptake of metals by plants. Selenium may also metal uptake through the modification of root morphology by the action of phytohormones^46,47^. The foliar application of nano-Se (25 mg L^−1^) clearly improved cucumber growth parameters such as plant height and leaf area, yield quality, chlorophyll content, plant biomass and enzymatic antioxidant capacities compared to other anti-stressor and control. Foliar Si application showed the greatest impact on enzymatic antioxidant capacities among the other anti-stressor treatments^48^.

## Conclusion

Therefore, if the purpose of producing *H.* *perforatum* is to use the shoots and essential oils of the plant, then the use of selenite and nano-selenium is not recommended. If the goal is to produce high hypericin, high hyperforin, and also the accumulation of selenium in the plant, treatments of 6 and 8 mg/l of selenium and nano-selenium could be used. A significant negative correlation between selenium content with morphological traits and yield of different organs and a positive correlation with the content of enzymes, chlorophylls, phenolic content, and proline shows that selenium causes stress in the plant and therefore plant growth is reduced and instead increases the production of antioxidant enzymes and osmolytes. There is a significant negative correlation between selenium content with morphological traits and function of different organs and a positive correlation with the number of enzymes, chlorophylls, phenolic content and proline indicates that selenium causes stress in the plant and therefore plant growth is reduced and instead production increases antioxidant enzymes and osmolytes.

## Abbreviations

S: Selenium
RWC: Relative water content
CTA: Catalase
POD: Peroxidase
MDA: Malondialdehyde
